# Practical Application of Aptamer-Based Biosensors in Detection of Low Molecular Weight Pollutants in Water Sources

**DOI:** 10.3390/molecules23020344

**Published:** 2018-02-07

**Authors:** Wei Zhang, Qing Xiu Liu, Zhi Hou Guo, Jun Sheng Lin

**Affiliations:** School of Medicine, Huaqiao University, Quanzhou 362021, Fujian, China; zw0915@163.com (W.Z.); 17011071006@hqu.edu.cn (Q.X.L.); 1601116005@hqu.edu.cn (Z.H.G.)

**Keywords:** aptamer, low molecular weight pollutant, water source, biosensor, environmental monitoring

## Abstract

Water pollution has become one of the leading causes of human health problems. Low molecular weight pollutants, even at trace concentrations in water sources, have aroused global attention due to their toxicity after long-time exposure. There is an increased demand for appropriate methods to detect these pollutants in aquatic systems. Aptamers, single-stranded DNA or RNA, have high affinity and specificity to each of their target molecule, similar to antigen-antibody interaction. Aptamers can be selected using a method called Systematic Evolution of Ligands by EXponential enrichment (SELEX). Recent years we have witnessed great progress in developing aptamer selection and aptamer-based sensors for low molecular weight pollutants in water sources, such as tap water, seawater, lake water, river water, as well as wastewater and its effluents. This review provides an overview of aptamer-based methods as a novel approach for detecting low molecular weight pollutants in water sources.

## 1. Introduction

Human activities, such as reckless industrial development, can result in serious environmental pollution with adverse effects on human health and animal lives, which have become a serious global issue [1,2]. Monitoring requirements of environmental contaminants are increasing [3]. Water sources must be strictly controlled to prevent their pollution with hazardous substances [4,5]. Most of the harmful substances in water sources are low weight molecules less than 1000 Da of formula weight [6]. They are non-immunogenic, so antibodies are inappropriate as recognition receptors for the purpose of monitoring. In addition, almost of the existing wastewater treatment plants were not traditionally designed to remove these low molecular weight pollutants, therefore contribute to the introduction of different levels of a variety of low molecular weight pollutants from different plants by different ways into the environment [7,8,9]. Sewage treatment plant effluents and wastewater discharges are considered to be a major source of low molecular weight pollutants like endocrine disrupting chemicals (EDCs) that are directly released into the aquatic environment [10,11]. There is an urgent need for the development and innovation of monitoring systems, which should be sensitive, quick, specific, inexpensive and convenient for users to monitor the quality of treated wastewater effluents as well as the natural water sources. Up to now, numerous analyzing techniques including spectroscopic, chromatographic, and electrochemical technologies have been employed for environmental monitoring. Although these technologies are fairly sensitive and accurate when operated by professionals, most of them are expensive, time-consuming, cumbersome, and/or require sample pretreatment [3,12,13,14,15,16]. In addition, these complex analyses have also limited to on-site and real-time detecting and monitoring the low molecular weight pollutants in water sources. Thus, the detection of low molecular weight pollutants and, if possible, their removal is of utmost importance [4]. 

In recent years, aptamers as chemical antibodies, have attracted significant interests and their applications in environmental monitoring systems have been continuously studied [17,18]. Aptamers are single-stranded nucleic acids, RNA or single-stranded DNA (ssDNA). They can non-covalently bind to a target molecule, including a broad range of targets (e.g., small molecules, ions, proteins, cells, tissues and organisms) with high affinity and specificity similar to antigen–antibody interactions [19,20,21]. The molecular recognition of aptamers and their target are based on molecular shape complementarities, stacking of aromatic rings, electrostatic or van der Waals interactions, and hydrogen bonding [22]. Aptamers, first introduced by three groups independently in 1990 are selected via an in vitro process known as the Systematic Evolution of Ligands by EXponential enrichment (SELEX) [23,24,25]. Aptamers can be selected from a random ssDNA or RNA library (usually 10^15^~10^16^ different sequences) by means of three main steps including selection, separation, and amplification. Based on their characteristics, aptamers can be used in different applications including target detection [26,27,28,29]. Compared to the generation of antibodies, SELEX processes allow greater control over binding conditions, and allows selection under non-physiological conditions [4,30]. In addition, DNA has higher stability and tolerance in different physical and chemical conditions than proteins (e.g., high temperature or extreme pH) [31,32]. How to generate aptamers for low weight molecules has been well reported or reviewed recently by Yang et al [33], Pfeiffer and Mayer [4], and Kin and Gu [30]. These oligonucleotide probes offer several advantages over the traditional antibodies, such as reusability, stability, and lack of immunogenicity and toxicity. In addition, ease of synthesis and modification makes them ideal for recognition of low molecular weight pollutants in most biological and environmental samples including water samples [34].

In recent years, aptasensors (aptamer-based biosensors [35]) have been applied for detection of low molecular weight pollutants in water sources including tap water, seawater, lake water, river water, as well as the wastewater and its effluences, contributing to improve monitoring for water quality. Articles about detection of low molecular weight pollutants in water samples using aptasensors or other sensors in last six years (since 2012) are selected for this review. This article outlines the most recent applications of aptasensors in water sources for detection of low molecular weight pollutants such as heavy metal ions, low weight molecular toxins, EDCs, drugs and pesticides. 

## 2. Aptasensors and Their Applications for Detecting Low Molecular Weight Pollutants in Water Sources

### 2.1. Types of Aptasensors

Aptasensors, namely aptamer-based biosensors, utilize aptamers for selective molecular recognition coming with a variety of readout mechanisms [4,35]. Recently, unique recognition capability of aptamers has attracted more attention to scientists because of its rapid response, high sensitivity and easy fabrication [36,37].

#### 2.1.1. Fluorescence-Based Aptasensors

Fluorescence detection is widely employed in bioanalytical chemistry including aptamer-ligand interactions [38]. Aptamers can be easily conjugated with several conventional fluorophores and quenchers. Thus, this type of aptasensor is more suitable for real-time detection [38]. The highly flexible structure of aptamers is suitable for construction of different types of fluorescent sensing devices, including molecular beacons, duplex structure with complementary sequences, structure switching, and competitive laser-based lateral flow assays [39,40,41,42]. An increasing number of fluorescence-base aptasensors has been used for monitoring low molecular weight pollutants in water sources, such as graphene oxide (GO)-based fluorescence resonance energy transfer (FRET) assay [43], magnetic bead composites coated with gold nanoparticles (AuNPs) and nicking enzyme fluorescence assay [44], carbon nanotubes (CNTs)-based fluorescent assay [45,46], biocompatible graphene quantum dots (QDs)-based fluorescent nanosensor [47]. 

#### 2.1.2. Colorimetric Aptasensors

Colorimetric assays are very attractive methods because they can be handily performed and their result signals can be measured and determined easily by the naked eye [48]. Most colorimetric assays are based on the principle that the visible color of AuNP suspensions varies with the change of dispersion-aggregation status. The aptamers are placed on the surface of AuNPs to prevent them from aggregation via electrostatic repulsion of aptamers. In presence of their target, the aptamers detach out from the AuNPs surface to bind their own targets inducing the nanoparticle aggregation thereby changing its visible color [49,50]. In addition, other materials are also used in colorimetric aptasensor including peroxidase-like activity of graphene/nickel@palladium hybrids [51] and hemin-functionalized reduced graphene oxide (hemin-rGO) composites [52].

#### 2.1.3. Electrochemical Aptasensors

Fast and simple electrochemical analysis is another attractive measurement method for application in biosensors with portable and low-cost detection. Recently, aptamers as recognition elements have been introduced in electrochemical sensing platform for low weight molecules [53,54,55]. Among them, label-free electrochemical impedance spectroscopy (EIS) as a powerful and sensitive technique to monitor aptamer-ligand binding has appeared as a promising strategy [56]. More importantly, EIS method is nondestructive, which makes it highly attractive for aptamer-based low weight molecules detection [57]. In addition, some novel methods using microwires formed by platinum nanoparticles (PtNPs) [58], AuNPs dotted rGO nanocomposite [59], thionine–graphene nanocomposites [60] are developed for detecting low molecular weight pollutants in water samples recently.

#### 2.1.4. Some Other Portable Aptasensors

Some portable aptasensors are based on urease/glucoamylase-trapped aptamer-cross-linked hydrogels. After addition of the targets, the aptamers bind to their target with high affinity thereby dehybridizing from the complementary strands, causing the collapse of the hydrogel network and resulting in the release of urease or glucoamylase into the solution. The enzyme then hydrolyzes the urea (pH changes) or sucrose (glucose increases), these concentration-dependent changes can easily be measured using portable pH meters or glucometers [61,62,63]. In addition, other portable aptasensors using smartphones as readout [64,65], optic fiber technology [66], and microchannels coupled with portable analyzer [67] have also been developed for on-site monitoring the pollutants in water samples in recent years.

Undoubtedly, more and more research and development on aptasensors will be conducted worldwide due to various advantages of aptamer-based recognition elements. One of the promising areas for aptasensors in detection of low molecular weight pollutants is in the water environment, since it is difficult to produce antibodies against low molecular weight pollutants with none or low immunogenicity [68]. Further development of mass-based aptasensors will depend on new amplificating signal techniques, in order to detect low molecular weight pollutants with high sensitivity and selectivity in water sources. 

### 2.2. Applications of Aptasensors in Detection of Low Molecular Weight Pollutants in Water Sources

**Figure 1:**
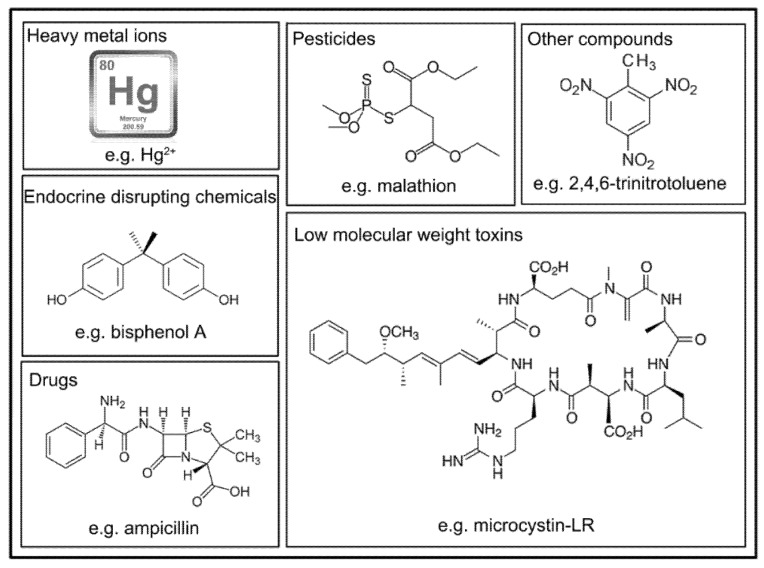
Chemical Structures of Representative Pollutants.

In recent years, application of aptasensor in detecting low molecular weight pollutants in environmental or food samples, but not in water sources, have been well reviewed [4,6,30]. In fact, aptasensors based on different principles have been developed to detect low molecular weight pollutants in water sources, such as toxins, hormones, drugs, and pesticides in water sources (Figure 1 and Table 1). 

#### 2.2.1. Detection of Heavy Metal Ions in Water Sources 

Heavy metals are widespread and persistent pollutants with our great concern as they are non-degradable, highly toxic and ubiquitous [118]. They are released into water bodies as a result of human activities mainly including industries and agriculture [119]. Heavy metals tend to be accumulated in animals and humans through a variety of food chains. Long-term exposure will lead to serious health problems in organisms including the disorders in the central nervous and reproductive systems, and even radiation carcinogenic effects [120,121]. In recent years, many authors have reviewed the methods based on aptamers for the detection of heavy metal ions, but hard to find a review that is special for water sources [63,122]. Therefore, it is imperative to promote the research progress for aptasensors to detect heavy metal ions in water bodies.

Its poisoning of Minamata Bay of Japan widely activated a global attention to mercury (Hg^2+^) toxicity and its potential consequences to the aquatic ecosystem and human health [123]. Currently, many types of aptasensors were developed for detecting the Hg^2+^ in water samples based on the different platforms, including electrochemistry, colorimetric and fluorescence-based analysis [73,74,75]. In addition, many signal amplification modes or novel platforms such as chemiluminescence (CL) [73], electrically rGO-based chemiresistor [69], silicon nanomaterials (SiNPs)-based fluorescence [70], fluorescence lateral flow strip [40] and exothermic chip [71] have also been developed for detecting the Hg^2+^ in water samples. Among those, a simple and rapid CL aptasensor for Hg^2+^ was developed in natural water samples with a limit of detection (LOD) of 16 pM within 30 min. This aptasensor was based on the principle of AuNPs charge effects and aptamer conformation change (from free state to T-Hg^2+^-T complex) induced by target to stimulate the generation of CL in the presence of H_2_O_2_ and luminol [73].

In addition, an electrochemically rGO-based chemiresistive aptasensor was assembled for detection of Hg^2+^ in tap and river water samples with good recovery rates (98.6–111.9% for tap water and 90.5–116.7% for river water), it obtained a LOD of 0.5 nM in the presence of other metal ions and matrices [69]. Currently, an optical biosensor based on the structure-switching DNA aptamer with in situ/on-site detecting of Hg^2+^ in natural water samples. This aptasensor is based on the selectively binding capacity of Hg^2+^ to T-T mismatch structure (fluorescence-labeled aptamer containing T-T mismatch structure) to form stable T-Hg^2+^-T complexes and then the fluorescence labeled-aptamer dehybrided from the sensor surface, which leads to decrease in fluorescence signal. A LOD of 1.2 nM was obtained in less than 10 min, which is below the standards set by the different governmental agencies in drinking water [72,123]. The specificity of the sensing system was evaluated for other potential interfering metal cations such as Mn^2+^, Mg^2+^, Ca^2+^ and the sensor exhibits high specificity toward Hg^2+^ [72]. The main advantages of this biosensor include sensitivity, portability, speediness, and minimum sample preparation.

Due to the adverse impacts of Hg^2+^ on human health, many biosensors have been developed to detect Hg^2+^. For examples, a DNA probe-based optical biosensor with the principle of integration of Hg^2+^-mediated T-T stabilization for detecting Hg^2+^ with a LOD of 0.027 nM and good recoveries of 92.5–112.0% in samples of bottle water and tap water [124]. Another DNA-protein conjugates-based optical fiber biosensor using the same principle of T-T mismatches for detecting Hg^2+^ obtained a LOD of 1.06 nM in a facile way [125]. In addition, a peptide nucleic acid (PNA)-based electrochemical biosensor was described for the determination of Hg^2+^ with high selectivity. The linear response towards Hg^2+^ was in the range from 5 to 500 nM with a LOD of 4.5 nM [126]. Moreover, a Hg^2+^-specific oligonucleotide-based electrochemiluminescent (ECL) biosensor for the detection of Hg^2+^ was easily prepared with a LOD of 5.1 pM [127]. In addition, a functional nucleic acid (FNA)–based fluorescent biosensor was developed for detecting Hg^2+^ and Pb^2+^ in water samples. The Hg^2+^ and Pb^2+^ could be selectively detected as low as 3.23 ppb and 2.62 ppb, respectively [128]. As discussed above, almost biosensors for Hg^2+^ detection are designed using recognition elements of nucleic acids including aptamers, DNA-protein conjugates and FNA. Of those, aptamers are more stable due to they are shorter than other nucleic acids recognition elements. Thus aptasensors require more development to detect Hg^2+^ in water.

Lead (Pb^2+^) is a highly poisonous metal (whether inhaled or swallowed), affecting almost every organ in the human body [129]. An exothermic chip-based aptasensor was developed for quantitative testing of Pb^2+^ using a forehead thermometer as readout. In this system, LOD was quantitatively analyzed as 0.045 μM, and good recoveries were evaluated in different water samples (mineral drinking water, purified drinking water and tap water) [71]. In addition, a photoluminescent graphene oxide quantum dot (GOQD) aptasensor exhibited highly selective and sensitive Pb^2+^ detection with a LOD of 0.64 nM and a dynamic range from 1 to 1000 nM. For testing this aptasensor in water samples , good recoveries of 82.0% to 116.4% were obtained in drink water, tap water and lake water [76]. Moreover, a graphene-based electrochemical aptasensor for Pb^2+^ was constructed for detecting Pb^2+^ in river water and tap water. Its detection limit was estimated to be 0.032 pM [77].

Other biosensors are also developed for detecting Pb^2+^ in water samples. For examples, a three-dimensional (3D) origami electrochemical device for sensitive Pb^2+^ testing based on DNA functionalized iron-porphyrinic metal-organic framework. This method showed detecting performance of Pb^2+^ concentration ranging from 0.03 to 1000 nM with a LOD of 0.02 nM [130]. In addition, a Pb^2+^-specific DNAzyme-based biosensor showed high sensitivity and selectivity with a LOD of 2 pM, providing potential application for Pb^2+^ detection in contaminated sewage and spiked drinking water samples [131]. Some sensing platforms using Pb^2+^ specific DNAzyme base on quartz crystal microbalance with dissipation (QCM-D) [132] and ZnO nanoflower (NF) photoelectrochemical (PEC) analysis [133] are also used in detecting Pb^2+^ in water samples. In comparison with enzymes and antibodies, DNAzymes and aptamers have remarkable advantages [31]. As mentioned above, aptamers and DNAzymes are mainly used for detecting Pb^2+^ in water sample, thus nucleic acids-based biosensors can be further developed for Pb^2+^ pollutant monitoring.

Arsenic (As^3+^) is a potent cardiovascular toxicant associated with numerous biomarkers of cardiovascular diseases in exposed human populations [134]. Currently, As^3+^ contamination of drinking water has been a single most important public health issue in Bangladesh [135]. Thus, monitoring and detection of As^3+^ in water sources are urgently needed. Up to date, several biosensors have been proposed for solving this problem. For examples, a “signal-on” screen-printed carbon electrode (SPCE)-based electrochemical aptasensor was fabricated for highly sensitive and selective detection of As^3+^. In this system, As^3+^-specific aptamer could adsorb cationic poly diallyldimethylammonium (PDDA) via electrostatic interaction to repel other cationic species. In the presence of targets, formation of aptamer/As^3+^ complex led to less adsorption of aptamers on PDDA, then produced a sensitive “turn-on” response. This proposed aptasensor exhibited a LOD of 0.15 nM. An excellent specificity was evaluated against other cations and good recoveries of 96.2–117.5% were obtained from tap and lake water [79]. The main advantages of this aptasensor are low cost and simple fabrication [79]. In addition, a field-effect transistor (FET)-type aptasensor using molybdenum disulfide (MoS2) nanospheres for As^3+^ detection showed an unprecedentedly detection (ca. 1 pM) with extraordinary responses (less than 1 s). The aptasensor also could discriminate target analytes in river water samples [78]. Moreover, AuNPs-based colorimetric aptasensor using cationic polymers was performed for the detection of As^3+^ in aqueous solution with the LOD of 5.3 ppb [82]. A novel SERS-based aptasensor based on Au@Ag core-shell nanoparticles was developed for detection of As^3+^ with detection limit of 0.1 ppb [83]. 

As^3+^ contamination has also been detecting by other types of biosensors recently. For examples, a low cost color-based bacterial biosensor was developed for measuring As^3+^ in groundwater. The bacterial biosensor demonstrates a quantitative range from 10 to 500 μg·L^−1^ of As^3+^ in 3 h reaction time. The shelf life for this biosensor is about 9 days in 4 °C and 3–5 days at room temperature [136]. In addition, biosensors including thermoresponsive magnetic nanobiosensors using green fluorescent protein-tagged sensor proteins [137], metallothionein-based biosensor [138], and multicolor fluorescent sulphur doped carbon dots-based sensor [139] are also used for detecting As^3+^ in water sources. Most of the biosensors for detecting As^3+^ in water sources are using either proteins or aptamers as a recognition element. As compared to aptamers, more limitations are found in proteins as recognition modules in biosensor applications. Thus, to a certain extent, aptasensors are more suitable for development for detection As^3+^ in water samples in the future.

Recently, some aptasensors are developed for detection of multiple heavy metal ions. For example, an extensible, facile and sensitive multidimensional sensor based on DNA-gold nanoparticle (DNA-AuNP) conjugates was developed for heavy metal ions (Ag^+^, Hg^2+^, Cr^3+^, Sn^4+^, Cd^2+^, Cu^2+^, Pb^2+^, Zn^2+^, and Mn^2+^) discrimination. A highly sensitive discrimination of metal ion targets with the detection limit as low as 50 nM with 100% identification accuracy is obtained [75]. The advantage of the multidimensional sensor is that it can improve the ability of target recognition just by adding a suitable sensing element.

#### 2.2.2. Detection of Low Molecular Weight Toxins in Water Samples

Environmental toxins produced by cyanobacteria and dinoflagellates have increasingly become a public health concern due to their damaging effects on the tissues in humans [140]. Several marine microalgae produce dangerous toxins harm to human health, aquatic ecosystems and coastal resources [141]. For example, microcystins are released by cyanobacteria in aquatic system of eutrophication. They contaminate grains like wheat and grain-derived products such as beer or baby food. They can also be found in milk, wine or coffee. Therefore, reliable early detection systems for such toxins need to be developed [142]. 

Exposure to microcystins is a global health problem because of its association with various other pathological effects. Methods for monitoring of this kind of toxins in water sources are very important. A simple, sensitive and selective aptamer-based colorimetric sensor was applied for detection of microcystin-LR (MC-LR), one of the most poisonous microcystins, using AuNPs sensing materials. In this system, a LOD of 0.37 nM with a range from 0.5 nM to 7.5 μM was obtained. In addition, lower signal responses to other toxins such as acetaminprid, glyphosate, dylox, atrazine and clofentezine were observed showing a good specificity [86]. Testing the feasibility of this aptasensor in real sample, good recoveries (95.0–102.2%) were obtained in tap and pond water samples [86]. Another aptamer-antibody immunoassay (AAIA) was developed for detecting MC-LR by a photodiode-based portable analyzer less than 35 min. In this aptasensor, LOD was 0.3 μg·L^−1^. A high specificity for MC-LR was detected against MC-LA, MC-YR, or nodularin-R and a good recovery of 94.5% to 112.7% was calculated in two spiked environmental samples [67]. In addition, AuNF/AgNPs-based aptasensor using SERS sensing platform were fabricated for detecting MC-LR. This aptasensor achieved the sensitive detection of MC-LR in lake water samples with the LOD of 8.6 pM [88]. Moreover, an upconversion nanoparticles (UCNP) grafted MoS_2_ nanosheets–based fluorescence aptasensor was used for MC-LR sensing. This assay specifically determined MC-LR in the linear range of 0.01–50 ng·mL^−1^ with a LOD of 0.002 ng·mL^−1^. This aptasensor obtained the recoveries of 94–112% in tap and lake water samples [87].

Other biosensors are also used for monitoring the MC-LR in water samples. For example, a 3D graphene-based immunosensor was developed for MC-LR quantification. A very good linear correlation of the electron-transfer resistance was achieved over 0.05 and 20 mg·L^−1^. Also, LOD of 0.05 mg·L^−1^ was accomplished [143]. In addition, a MoS_2_/gold nanorod composite-based electrochemical immunosensor was developed for sensitive and quantitative detection of MC-LR in lake, tap and drinking water samples. Under optimal conditions, the immunosensor exhibited a linear response to MC-LR ranging from 0.01 to 20 μg·L^−1^ with a detection limit of 5 ng·L^−1^. The recovery of 99.7% to 102.1% was obtained by this biosensor [144]. Furthermore, a novel recombinant protein phosphate type 1 (PP1α)-based electrochemical MC-LR biosensor is reported. This biosensor exhibited a LOD at 0.93 μg·L^−1^ with significant selectivity and sensitivity towards MC-LR [145]. The biosensors using aptamers, antibodies and recombinant protein are involved in detecting MC-LR in water samples. Due to the advantages of aptamers over proteins, aptasensor are more suitable for MC-LR monitoring in natural water samples.

Saxitoxin (STX), one of potent neurotoxins produced by marine dinoflagellates, is well known for its role in acute paralytic poisoning by preventing the generation of action potentials in neuronal cells [146]. To detect the STX in water samples, a label-free and real-time optical biolayer interferometry (BLI) competitive aptasensor has been employed. The biosensor exhibited a broad detection ranges from 10 to 2000 ng·mL^−1^ of STX, with a low detection limit of 0.5 ng·mL^−1^. In addition, another potent neurotoxin, anatoxin-a (ATX), is also need to be monitoring [84]. For example, a label-free impedimetric aptasensor was developed for ATX detection. The aptasensor showed a LOD of 0.5 nM and a linear range between 1 and 100 nM. This aptasensor have been tested with recoveries of 94.8–108.6% in drinking water samples [84]. Recently, methods for detection of ATXs in water samples are still using the conventional technical methods such as ion mobility spectrometry [147], liquid chromatography-quadrupole time-of-flight high resolution mass spectrometry [148], diode thermal desorption-atmospheric pressure chemical ionization (LDTD-APCI) coupled to the Q-Exactive [149], and LC-MS/MS [150]. 

Recently, efforts are also done on the detection of marine biotoxin palytoxin. For example, an enzyme-linked aptamer coupled with BLI biosensor was designed for detection of palytoxin. In this system, horseradish peroxidase (HRP)-labeled aptamers were immobilized on the biosensor surface to competitively bind with palytoxin. After submerging in a 3,3’-diaminobenzidine (TMB) solution leads a large change in the optical thickness of the biosensor layer. This change could obviously shift the interference pattern and generate a response profile on the BLI biosensor. The biosensor showed a broad linear range for palytoxin (200 to 700 pg·mL^−1^) with a low detection limit (0.04 pg·mL^−1^) [91]. The aptasensor are features as real-time, ultra-sensitivity, and rapid test.

#### 2.2.3. Monitoring of Endocrine Disrupting Chemicals in Water Samples

EDCs are environmental micropollutants (natural or anthropogenic) that alter the function of the endocrine system, by interfering with hormone biosynthesis, metabolism, or action, and consequently causing disturbances in the endocrine system even cause increased incidence of cancers [10]. Nearly 800 chemicals are known to have more or less interference effects on endocrine system [151]. It has been alerted regarding the potential adverse effects of EDCs on health of human and wildlife [152]. Although the presence of endocrine disruptors in water sources is usually very low, EDCs have been constantly released and spread into our daily environment, and still are going on [10].

Bisphenol A (BPA), one of EDCs, usually exists in daily plastic products, and is one of the most serious environment contaminants [153]. BPA was detected in water by aptasensors based on AC electrokinetics (ACEK) capacitive sensing method successfully at femtomolar (fM) levels within 30 s in water samples [154]. The sensor was responsive to BPA down to 1 fM with a range of 1.0 to 10 fM, but not to structurally similar compounds, including BPF or BPS, even at much higher concentration [154]. In addition, a portable, evanescent, wave fiber-optic aptasensor was developed for on-site detection of BPA quickly with good recoveries of 91.7–110.4% in tap water and wastewater samples. The developed aptasensor could detect BPA in 10 min and exhibited a range of 2 to 100 nM with a low detection limit of 1.86 nM (0.45 ng·mL^−1^) [66]. This aptasensor can also be reused by SDS solution and do not need any pre-treatment of water samples. Furthermore, another universal and sensitive graphene oxide (GO)-based fluorimetric aptasensor was developed for detecting BPA in tap water with good recoveries of 95.0–105.0%. It obtained a LOD of 0.005 μg·L^−1^ with a detection range of 0 to 1.0 μg·L^−1^ [101]. Moreover, an AuNPs-based fluorescence aptasensor was performed to detect the BPA in water sample and obtained a LOD at 0.1 ng·mL^−1^ [102]. Another magnetic GO-based fluorescent aptasenor for detecting BPA has also been applied in water samples. LOD of this aptasensor was 0.071 μg·L^−1^, range from 0.2–10 μg·L^−1^. The biosensor exhibited excellent anti-interference ability and selectivity in actual water samples, testing by HPLC and this aptasensor [103]. In addition, an exceptional electrochemical aptasensor using the 3D conductivity and hierarchical porous structure was used for BPA detection. This aptasensor obtained a LOD of 0.33 nM and a range from 10 nM to 1 mM with good recoveries of 94.0–108.0% in natural lake water samples [100]. 

Other biosensors are also performed for BPA detection in water samples. For examples, a label-free DNA Y junction sensing platform for amplified detection of BPA using exonuclease III-based signal protection strategy has been reported. LOD of this system was 5 fM without any labeling, modification, immobilization or washing procedure [155]. An electrochemical biosensor based on a diazonium-functionalized boron-doped diamond electrode modified with a multi-walled CN-tyrosinase hybrid film was developed for BPA detection. The biosensor displayed a large linear range from 0.01 to 100 nM, with a LOD of 10 pM [156]. In addition, a reusable evanescent wave immunosensor has been employed for highly sensitive detection of BPA in water samples. Typical calibration curves showed a detection limit of 0.03 μg·L^−1^ for BPA [157].

Some of the EDCs that are introduced to the environment can be naturally generated by hormone 17β-estradiol (E2). It has been known that E2 via the estrogen receptor α elicits rapid signals driving breast cancer cells to proliferation. Currently, a great process for E2 biosensors has made to detect its concentration in water. An ultrasensitive PEC aptasensor was applied in water analysis with a LOD of 33 fM. And the analytical results of the aptasensor in water samples showed good agreements with that determined by HPLC [93]. An optical aptasensor was developed for rapid and sensitive detection of E2 in wastewater effluents. Its LOD was 2.1 nM (0.6 ng·mL^−1^) with good recoveries of 94.1–104.8%. It is also can be reused by 0.5% SDS solution (pH 1.9) over tens of times [95]. In addition, aptamers for detection of E2 with dissociation constant of 0.6 μM was selected and tested in water samples including laboratory water, lake water and tap water, which had a good sensitivity and selectivity toward to targets in natural water samples [94]. A smartphone imaging-based label-free and dual-wavelength fluorescent aptasensor for detecting E2 in wastewater samples has been reported. The LOD was 1 fg·L^−1^ testing in wastewater [96].

Antibody-based biosensors are also investigated for E2 detection in water samples recently. However, the utilization of any antibody-based device is limited by the intrinsic characteristics of protein, such as temperature for storage and transport and working conditions for application. A good biosensor based on antibody was demonstrated lasting 14 days with 70% of initial response [158]. Other immunosensors, including differential pulse voltammetry (DPV) immunosensors [159] and double-layer molecularly imprinted film-based biosensors [160] were also developed for detecting E2 in water samples such as waste seawater effluents. 

A label-free impedimetric aptasensor was developed for detecting progesterone (P4) in tap water samples. The aptamer obtained a range of detection from 10 to 60 ng·mL^−1^ with a detection limit of 0.90 ng·mL^−1^. Moreover, the aptasensor was applied in spiked tap water samples showing good recovery percentages [107]. In addition, a highly selective fluorescence-based p4 aptasensor was developed for detecting P4 in tap water samples. A linear relationship of this P4 aptasensor was obtained in the range from 10 to 100 ng·mL^−1^ and the detection limit was calculated to be 110 pg·mL^−1^ [42]. 

#### 2.2.4. Detection of Drugs in Water Samples

Antibiotics are used in the treatment of bacterial infections, their partial synthetic derivatives or chemically synthesized compounds are largely used in livestock industry and agricultural production. They have also been applied in both veterinary and human medicine. The widespread use of antibiotics has led to a series of problems in the aquatic environment. Furthermore, globally occurrences of bacterial resistance affects almost all of the bacterial pathogens and epidemiological settings [161]. The aptamer-based biosensors for detection of antibiotic have been well reviewed by Katrin and Gerd [161].

Recently, a label-free and user-friendly fluorescence-based aptasensor was developed for monitoring ampicillin in river water samples using magnetic bead composites coated with AuNPs and a nicking enzyme. The LOD of this aptasensor was 0.07 ng·L^−1^ with a recovery of 90.0%–120.0% in polluted river water [44]. An AuNPs-based dual fluorescence-colorimetric aptasensor has been developed to detect ampicillin in milk sample. The lower LOD was 2 pg·L^−1^ by fluorescence and a 10 pg·L^−1^ by colorimetry tested in milk [162].

An aptasensor using the streptavidin plates combined with biotinylated aptamer labeled with fluorescence was performed to detect kanamycin A (KNA) in cleaned wastewater. The detection limit was 0.5 µM with the measuring range from 0 to 50 µM tested in water sample. The specificity was evaluated by analyzing other similar molecules (e.g., KNB, tobramycin, apramycin) and those display similarity to the selection target to a much lesser extent [109]. In addition, a simple and sensitive AuNPs-based fluorescence aptasensor was developed for the detection of KNA. The analytical linear range from 0.8 to 350 nM and the detection limit of 0.3 nM was realized successfully by this aptasensor. Good recovery rates from 99.2% to 105.1% in milk samples were also obtained [163]. 

Currently, a silver nanoclusters (AgNCs)-based fluorescent aptasensor was presented for detection of oxytetracycline (OTC). It obtained a LOD of 0.1 nM ranging from 0.5–100 nM, it also obtained good recoveries of 97.5–98.5% in tap water samples. The good selectivity of this aptasensor was evaluated by testing against the ampicillin, doxycycline and tetracycline [110]. In addition, a novel portable reflectance-based aptasensor using AuNPs for the detection of OTC in tap water samples has been reported. LOD of this aptasensor was detected at concentrations as low as 1 nM in both buffer solution and tap water. Specificity is evaluated by OTC, tetracycline, doxycycline, and diclofenac [111]. For immunosensors, antibody-based paper strips were applied to the analysis of spiked environmental water, allowing a quantitative determination for OTC concentrations as low as 30 ng·mL^−1^ [164].

A sensitive, rapid and label-free colorimetric aptasensor for sulfadimethoxine (SDM) detection was developed based on the tunable peroxidase-like activity of graphene/nickel@palladium nanoparticle (Gr/Ni@Pd) hybrids. The detecting ranges were from 1 to 500 μg·L^−1^ with the detection limit of 0.7 μg·L^−1^. Good recoveries of 95.1–107.8% were also tested in natural lake water samples. The good selectivity was tested by evaluating the interfering substances like OTC, KNA, and sulfathiazole [51]. For other environmental samples, visible-light PEC aptasensor (LOD was 0.55 nM) [165] and coordination polymer nanobelt (CPNB)-based aptasensor (LOD was 0.55 nM) [166] were developed for detection SDM in veterinary drug formulation and milk, respectively. In addition, most of the commonly used non-prescription analgesics, which are harmful to fishes and birds, are also can be found in water source recent years [167]. These pollutants in water bodies are also need monitoring in time.

#### 2.2.5. Monitoring of Pesticides in Water Samples

Extensive use of pesticides and herbicides in agriculture, hence they are among the most important pollutants in water environment [168]. The organophosphorous insecticides are extensively used in agriculture, horticulture and household life. Dietary intake and other exposures to these high toxicity molecules have caused a major environmental concern [169]. 

Acetamiprid has been detected in water samples by a label-free electrochemical impedimetric aptasensor characterized by high selectivity, high sensitivity, and easily operation. With the aptasensor, a lower LOD was determined as 0.017 fM with a larger linear range from 0.05 fM to 0.1 μM. In that biosensor, AuNPs decorated multiwalled CNTs-rGO nanoribbon composite were used for conjugating the aptamer to electrode surface for signal amplification [112]. The selectivity of the aptasensor was also tested by determining interfering substances such as chlorpyrifos, imidadoprid, pentachlorophenol, methylparathion, carbaryl and the relative responses of the impedance were less than 4%, while it is 66% for acetamiprid, suggesting a good selectivity [112]. In addition, acetamiprid in water samples was detected using PtNPs-based impedimetric aptasensor [58] and resonance light-scattering (RLS)-based aptasensor [113], and obtained the LOD of 1 pM and 1.2 nM, respectively. These aptasensors are applied in natural lake, tap and the mineral waters to get good recoveries of 86.0% to 112.6%.

Nowadays, an AuNPs-based colorimetric aptasensor was developed for detecting malathion, an organophosphorus pesticide, found in contaminated lake water. The performance of this type of biosensor is quite simple and straightforward. It can be done completely in a few minutes without the need of any expensive equipment or trained personnel. It obtained a concentration range of 0.5–1000 pM with 0.06 pM as the LOD in optional buffer and obtained a good recovery of 88.0% to 104.0% in the lake water samples. The specificity of this aptasensor was evaluated against other oestucudes including atrazine, chlorosulfuron, 2,4 D, diuron, and phorate. Results showed that only the target malathion has the responses while the others are negligible [114]. In addition, a palladium-gold bimetallic nanozyme-based (as nanozyme) colorimetric biosensor was reported for detecting the malathion in tap water, it got a LOD of 60 ng·mL^−1^ with a good recovery of 80.0% to 106.0% in tap water [170].

A PtNPs-based aptasensor was also applied to detect the atrazine in water samples. It obtained a LOD of 10 pM with a good recovery of 79.0% to 113.0% in tap and mineral water samples. As compared to other biosensors, an AuNPs-based electrochemical immunosensor (antibody-based biosensor) for atrazine has been reported. LOD of 4.64 pM (0.001 ng·mL^−1^) was obtained with a good recovery of 87.3% to 108.3% in water samples (deionised water, riverine water, and seawater) [171]. In addition, a phage anti-immunocomplex electrochemical immunosensor (PhAIEI) was performed to detect the atrazine in river water samples. It obtained a LOD of 0.927 pM (0.2 pg·mL^−1^) with the good recovery of 96.0% to 99.0% in water samples [172]. 

As mentioned above, the main biosensor for trace pesticides monitoring are nanozyme-based, aptamer-based and antibody-based biosensors. Due to the limitations of antibodies, the aptasensors and nanozyme-based biosensors are more suitable for monitoring pesticides in real sample application in water sources [32]. Recently, multiple targets detection is a trend for environmental monitoring. An AuNPs-based colorimetric aptasensor was proposed to detect six organophosphorous pesticides including isocarbophos, phosalone, methamidophos, acephate, trichlorfon, and dursban. The proposed method was tested with these six pesticides in natural river water samples with good recoveries from 72.0% to 135.0% [115].

#### 2.2.6. Other Compounds in Water Sources

Accurate detection of commonly used explosive ingredients as well as their degradation products is essential for environmental protection and human safety. For example, a highly chemiluminescent magnetic beads and label-free sensor was used for detecting 2,4,6-trinitrotoluene (TNT) in natural river water samples with a good recovery from 90.0–108.0%. This sensor obtained a range from 0.05 to 25 ng·mL^−1^ with a LOD of 17 pg·mL^−1^. Moreover, its specificity was examined against four interfering compounds including NB, 2′NT, 3′NT and 2,4′DNT, they were showed a weak CL single response compared to the target of TNT [116]. For other compounds in water sources, an aptamer-based biosensor for label-free detection of ethanolamine by electrochemical (EA) impedance spectroscopy has been reported. This aptasensor obtained over the range of 0.16 to 16 nM EA, with a detection limit of 0.08 nM. Interference by other selected chemicals with similar structure was negligible (ethanol, glycol, isopropanolamine, and isopropanol) in this system [117].

There are still many problems that need to be overcome for field application of aptasensors. Most aptasensors have been demonstrated on spiked water samples, but not real unknown samples. There are rare exceptions, E2 in unspiked medical waste water (ranged from 0.2 to 10.90 pM for four hospital), unspiked lake water (ranged 0.58 from 1.87 pM for four different lake area) and unspiked tap water (below the LOD) were detected using aptasensor [93]. In addition, most of the aptasensors for low molecular weight pollutants are limited or applied inadequately in water samples. Therefore, further development of aptasensor for low molecular weight pollutants is need to focus on the specificity and adaptability in real water samples, especially in unspiked water samples.

## 3. Conclusions and Perspectives

Recently, many efforts discussed above have been made to realize high-performance detection of low molecular weight pollutants in water samples by different types of biosensors. Although antibodies offer high specificity corresponding to its antigen, some protein features limit the utilization of antibodies, such as poor immunogenicity for production (especially using low molecular weight pollutants as antigens to immunize animals), lower temperature requirement for storage and transportation, and short shelf life. Thus, from this review, we reach the conclusion that nucleic acids-based biosensors (e.g., aptamers, FNA and PNA) are the main directions for sensor development for water sources. Among those, aptasensors remain the most dominant biosensor. Most of the low molecular weight pollutants exist in very low concentrations in the water environment, even below the LOD of existing detection technologies. Some fields of the application of aptasensors or other sensors for low molecular weight pollutants in water sample are still needed to be further developed [173]. Here are some suggestions for aptasensors of application in real water sources in the future:

(1) Generate the aptamers with higher affinity for low weight molecules by modification of SELEX process and truncations of the original aptamers.
(2) Highly sensitive and selective analyses by developing more powerful signal amplification methods.
(3) On-site, long-period, remote monitoring and real-time analyses in water samples.
(4) Ultrafast, simple, label-free, cost-effective and multiplex analyses of complex water samples.
(5) Portable analyses through integration to miniaturized sensor elements.

Real water samples are much more complex than the solutions used in our laboratories, such as selection buffers. They frequently contain many potential signal interference molecules, which may cause non-specific interactions with the aptamers or false binding to the target molecules. In the future, affordable, practical, careful, rapid, sensitive, and efficient aptasensors or other detection methods need to be further developed for overcoming the limitation of low molecular weight pollutants detection in unspiked water samples. In addition, it is worth paying attention to isolate and remove low molecular weight pollutants from water sources by aptamer-based or other methods.

## Figures and Tables

**Table 1:** Summary of Aptasensor Types for Detecting Low Molecular Weight Pollutants in Water Samples (Since 2012).

Class	Target	Sensor Types	Limit of Detection	Chemistry	Sampling	Recovery (%)	Response Range	Year	Reference
Metals	Hg^2+^	Electrochemical	0.5 nM	DNA	Tap water River water	98.6–111.9% 90.5–116.7%	0.5 nM–990 nM	2016	[69]
Metals	Hg^2+^	Fluorescence	0.415 μM	DNA	Water	/	/	2015	[70]
Metals	Hg^2+^	Fluorescence	0.13 μg·L^−1^	DNA	River water	90.0–113.0%	0.13 μg·L^−1^–4 μg·L^−1^	2017	[40]
Metals	Hg^2+^	Others	0.045 μM	DNA	Mineral drinking water Purified drinking water Tap water	112.0% 104.0% 96.0%	0.1 μM –10 μM	2015	[71]
Metals	Hg^2+^	Fluorescence	1.2 nM	DNA	Natural lake water	/	0 nM–100 nM	2013	[72]
Metals	Hg^2+^	Colorimetric	16 pM	DNA	Tap water Lake water	96.3–98.9% 95.3–104.2%	0.62 nM–1.2 μM	2016	[73]
Metals	Hg^2+^	Electrochemical	0.0036 nM	DNA	River water Tap water Landfill leachate	100.5–100.6% 100.5–103.0% 100.7%	0.01 nM–5000 nM	2017	[74]
Metals	Hg^2+^	Colorimetric and Fluorescence	30 nM	DNA	River water	/	/	2016	[75]
Metals	Hg^2+^	Optical, smartphone based	0.28 μg·L^−1^	DNA	Tap water River water	93.0–113.0% 101.0–110.0%	1 μg·L^−1^–32 μg·L^−1^	2016	[64]
Metals	Cu^2+^	Fluorescence	1.5 μM	DNA	Water	/	1 μM–14 μM	2015	[70]
Metals	Cu^2+^	Colorimetric and Fluorescence	16 μM	DNA	River water	/	/	2016	[75]
Metals	Fe^2+^	Fluorescence	0.592 μM	DNA	Water	/	/	2015	[70]
Metals	Pb^2+^	Colorimetric and Fluorescence	0.24 μM	DNA	River water	/	/	2016	[75]
Metals	Pb^2+^	Others	0.081 μM	DNA	Mineral drinking water Purified drinking water Tap water	/	0.1 μM–10 μM	2015	[71]
Metals	Pb^2+^	Fluorescence	0.64 nM	DNA	Drink water; Tap water; Lake water	95.4–104.0% 82–116.4% 95.4–104.4%	1 nM–1000 nM	2015	[76]
Metals	Pb^2+^	Electrochemical	0.032 pM	DNA	River water Tap water	/	0.16 pM–0.1 nM	2016	[77]
Metals	Ag^+^	Colorimetric and Fluorescence	0.463 μM	DNA	River water	/	/	2016	[75]
Metals	Zn^2+^	Colorimetric and Fluorescence	15.3 μM	DNA	River water	/	/	2016	[75]
Metals	Cd^2+^	Colorimetric and Fluorescence	88.9 μM	DNA	River water	/	/	2016	[75]
Metals	Mn^2+^	Colorimetric and Fluorescence	1.8 μM	DNA	River water	/	/	2016	[75]
Metals	Cr^3+^	Colorimetric and Fluorescence	0.96 μM	DNA	River water	/	/	2016	[75]
Metals	Sn^4+^	Colorimetric and Fluorescence	0.667 μM	DNA	River water	/	/	2016	[75]
Metals	As^3+^	Field-effect transistor	1 pM	DNA	River water	/	1 pM–10 nM	2013	[78]
Heavy metal	As^3+^	Electrochemical	0.15 nM	DNA	Tap water Lake water	96.2–99.5% 107.3–117.5%	0.2 nM–100 nM	2016	[79]
Metals	As^3+^	Colorimetric and resonance scattering (RS)	40 ppb naked eye 0.6 ppb colorimetric 0.77 ppb RS	DNA	Water	Colorimetric 94.6–124.0% RS 94.6–117.0%	1 ppb–1500 ppb	2012	[80]
Metals	As^3+^	Resonance Rayleigh Scattering	0.2 ppb	DNA	Water	96.7–104.0%	0.1 ppb–200 ppb	2012	[81]
Metals	As^3+^	Colorimetric	5.3 ppb	DNA	Aqueous solution	/	/	2012	[82]
Metals	As^3+^	Surface-enhanced Raman scattering	0.1 ppb	DNA	Lake water	86.33–97.20%	0.5 ppb–10 ppb	2015	[83]
Toxins	Anatoxin-a	Electrochemical	0.5 nM	DNA	Drink water	94.8–108.6%	1 nM–100 nM	2015	[84]
Toxins	MC-LR	Colorimetric	0.5 ng·L^−1^–1 ng·L^−1^	RNA	Drink water	88.0 ± 3.0%	/	2012	[85]
Toxins	MC-LR	Colorimetric	0.37 nM	DNA	Tap water Pond water	95.0% 97.0–102.2%	0.5 nM–7.5 μM	2015	[86]
Toxins	MC-LR	Fluorescence	0.002 μg·L^−1^	DNA	Tap water and lake water	94.0–112.0%	0.015 μg·L^−1^–50 μg·L^−1^	2017	[87]
Toxins	MC-LR	Photodiode-based	0.3 μg·L^−1^	DNA	Lake water Pond water Tap water	110.9–112.7% 98.2–109.1% 94.5–107.5%	0.5 μg·L^−1^–4.0 μg·L^−1^	2014	[67]
Toxins	MC-LR	Surface-enhanced Raman scattering	8.6 pM	DNA	Lake water	94.48–97.70%	/	2015	[88]
Toxins	MC-LR	Electrochemical	0.04 μg·L^−1^	DNA	Tap water; distilled water; wastewater	/	0.1 μg·L^−1^–1.1 μg·L^−1^	2017	[89]
Toxins	Saxitoxin	Optical	0.5 μg·L^−1^	DNA	Sea water	101.4–105.5%	100 μg·L^−1^–800 μg·L^−1^	2017	[90]
Toxins	Palytoxin	Biolayer interferometry	0.04 ng·L^−1^	DNA	Sea water	100.27–105.04%	200 ng·L^−1^–700 ng·L^−1^	2016	[91]
Toxins	Cylindrospermopsin	Electrochemical	0.117 μg·L^−1^	DNA	Lake water	96.3–104.6%	0.39 μg·L^−1^–78 μg·L^−1^	2015	[60]
Toxins	Cylindrospermopsin	Electrochemical	100 pM	DNA	Tap water	95.8–103.2%	0.1 nM–80 nM	2014	[92]
EDCs	17β-estradiol	Photoelectrochemical	33 fM	DNA	Medical wastewater; lake water and tap water	/	0.05 pM–15 pM	2014	[93]
EDCs	17β-estradiol	Equilibrium filtration	0.6 μM ^a^	DNA	Laboratory; lake water and tap water	/	/	2015	[94]
EDCs	17β-estradiol	Fluorescence	2.1 nM	DNA	Wastewater effluent	94.1–104.8%	/	2012	[95]
EDCs	17β-estradiol	Fluorescence	1 fg·L^−1^	DNA	Wastewater	66.7–77.8%	1 fg·L^−1^–100 fg·L^−1^	2017	[96]
EDCs	17β-estradiol	Fluorescence	0.48 nM	DNA	Water	94.3–111.7%	0.48 nM–200 nM	2017	[97]
EDCs	17β-estradiol	Electrochemical	0.8 fM	DNA	Wastewater	93.6–100.2%	1 fM– 600 fM	2015	[98]
EDCs	17-α ethynylestradiol	Equilibrium filtration	0.5 μM–1.0 μM ^a^	DNA	Laboratory; lake water and tap water	/	/	2015	[94]
EDCs	BPA	Fiber-optic	1.86 nM	DNA	Tap and wastewater	91.7–110.4%	2 nM–100 nM	2014	[66]
EDCs	BPA	Probe and AC electrokinetics capacitive	1.0 fM	DNA	Water	/	1.0 fM–10 fM	2016	[99]
EDCs	BPA	Electrochemical	0.33 nM	DNA	Lake water	94.0–108.0%	10 nM–1 mM	2015	[100]
EDCs	BPA	Fluorescence	0.005 μg·L^−1^	DNA	Tap water	95.0–105.0%	0–1.0 μg·L^−1^	2017	[101]
EDCs	BPA	Fluorescence	0.1 μg·L^−1^	DNA	Water	/	1 μg·L^−1^–10,000 μg·L^−1^	2013	[102]
EDCs	BPA	Fluorescence	0.071 μg·L^−1^	DNA	Tap water; pure water; river water	/	0.2 μg·L^−1^–10 μg·L^−1^	2017	[103]
EDCs	BPA	Fluorescence	2 nM	DNA	River water	/	2 nM–20 nM	2017	[104]
EDCs	BPA	Photoelectrochemical	0.5 nM	DNA	Drinking water	96.2–108.4%	1 nM–1000 nM	2016	[105]
EDCs	BPA	Fluorescence	0.05 μg·L^−1^	DNA	Tap water River water	96.0–102.4% 97.2–104.5%	0.1 μg·L^−1^–10 μg·L^−1^	2015	[43]
EDCs	BPA	Surface-enhanced Raman scattering	10 fM	DNA	Tap water	/	10 fM–100 nM	2015	[106]
EDCs	PCB77	Electrochemical	0.01 μg·L^−1^	DNA	Tap water	/	0.2 μg·L^−1^–200 μg·L^−1^	2016	[57]
EDCs	PCB77	Colorimetric	0.05 nM	DNA	Pond water; river water	96.67–108.78%	0.5 nM–900 nM	2017	[50]
EDCs	PCB77	Electrochemical	0.1 pg·L^−1^	DNA	Tap water	/	1 pg·L^−1^–10 μg·L^−1^	2017	[59]
EDCs	Progesterone	Electrochemical	0.9 μg·L^−1^	DNA	Tap water	/	10 μg·L^−1^–60 μg·L^−1^	2015	[107]
EDCs	Progesterone	Fluorescence	110 ng·L^−1^	DNA	Tap water	88.6–95.2%	10 ng·L^−1^–100 ng·L^−1^	2017	[42]
Drugs	Sulfadimethoxine	Colorimetric	0.7 μg·L^−1^	DNA	Lake water	95.1–107.8%	1 μg·L^−1^–500 μg·L^−1^	2017	[51]
Drugs	Quinolones	Fluorescence	0.1 nM–56.9 nM ^a^	DNA	Sewage plant; wetlands and tap water	/	/	2015	[108]
Drugs	Ampicillin	Fluorescence	0.07 μg·L^−1^	DNA	Polluted river water	90.0–120.0%	0.1 μg·L^−1^–100 μg·L^−1^	2017	[44]
Drugs	Kanamycin A	Fluorescence	0.5 μM	DNA	Cleaned waste water	/	0–50 μM	2014	[109]
Drugs	Oxytetracycline	Fluorescence	0.1 nM	DNA	Tap water	97.5–98.5%	0.5 nM–100 nM	2015	[110]
Drugs	Oxytetracycline	Colorimetric	1 nM	DNA	Tap water	/	0–5 nM	2015	[111]
Pesticides	Acetamiprid	Electrochemical	0.017 fM	DNA	Water	96.0–106.6%	0.05 fM–0.1 μM	2015	[112]
Pesticides	Acetamiprid	Resonance light-scattering	1.2 nM	DNA	Lake water	92.2–112.6%	0–100 nM	2016	[113]
Pesticides	Acetamiprid	Electrochemical	1 pM	DNA	Tap water Mineral water	86.0–102.0% 106.0–112.0%	10 pM–100 pM	2017	[58]
Pesticides	Malathion	Colorimetric	0.06 pM	DNA	Lake water	88.0–104.0%	0.5 pM–1000 pM	2016	[114]
Pesticides	Isocarbophos	Colorimetric	/	DNA	River water	72.0%	/	2015	[115]
Pesticides	Phosalone	Colorimetric	/	DNA	River water	135.0%	/	2015	[115]
Pesticides	Methamidophos	Colorimetric	/	DNA	River water	123.0%	/	2015	[115]
Pesticides	Acephate	Colorimetric	/	DNA	River water	89.0%	/	2015	[115]
Pesticides	Trichlorfon	Colorimetric	/	DNA	River water	78.0%	/	2015	[115]
Pesticides	Dursban	Colorimetric	/	DNA	River water	80.0%	/	2015	[115]
Pesticides	Atrazine	Electrochemical	10 pM	DNA	Tap water Mineral water	79.0–99.0% 106.0–113.0%	100 pM–1 μM	2017	[58]
Others	TNT	Chemiluminescent	17 ng·L^−1^	Peptide	River water	90.0–108.0%	0.05 μg·L^−1^–25 μg·L^−1^	2017	[116]
Others	Ethanolamine	Electrochemical	0.08 nM	DNA	Tap water	/	0.16 nM–16 nM	2016	[117]

Notes: ^a^ it means dissociation constant.

## Abbreviation

ATX Anatoxin-a; AuNPs Gold nanoparticles; BLI Biolayer interferometry; BPA Bisphenol A; CL Chemiluminescence; CNTs Carbon nanotubes; DPV Differential pulse voltammetry; E2 17β-estradiol; EDCs Endocrine disrupting chemicals; ECL Electrochemiluminescent; EIS Electrochemical impedance spectroscopy; FET Field-effect transistor; FNA Functional nucleic acid; FRET Fluorescence resonance energy transfer; GOQD Photoluminescent graphene oxide quantum dot; HRP Horseradish peroxidase; KNA Kanamycin A; LOD Limit of detection; MC-LR Microcystin-LR; MoS_2_ Molybdenum disulfide; NF Nanoflower; OTC Oxytetracycline; P4 Progesterone; PEC Photoelectrochemical; PNA Peptide nucleic acid; PtNPs Platinum nanoparticles; PCB 77 Polychlorinated Biphenyl 77; QDs Quantum dots; RLS Resonance light-scattering; QCM-D Quartz crystal microbalance with dissipation; SELEX Systematic Evolution of Ligands by EXponential enrichment; ssDNA Single-stranded DNA; SDM Sulfadimethoxine; SERS Surface-enhanced Raman scattering; SiNPs Silicon nanomaterials; STX Saxitoxin; T-Hg^2+^-T Thymine-Hg^2+^-thymine; T-T mismatch structure Thymine-thymine mismatch structure; TNT 2,4,6-trinitrotoluene; UCNP Upconversion nanoparticles; 3D Three-dimensional.
